# Bergamot Leaf Extract as an Agent Against Chronic Liver Diseases? In Vitro and In Vivo Findings on Oxidative Stress Modulation

**DOI:** 10.3390/antiox14050543

**Published:** 2025-04-30

**Authors:** Taynara Aparecida Vieira, Danielle Gabriel Seloto, Joyce Santana Rizzi, Paloma Vitória Lima Peixoto, Giulia Vitória Betoni Corrêa, Juliana Silva Siqueira, Nubia Alves Grandini, Erika Tiemi Nakandakare-Maia, Letícia Cardoso Valente, Fabiane Valentini Francisqueti-Ferron, Artur Junio Togneri Ferron, Giovanna Baron, Giancarlo Aldini, Camila Renata Correa, Lilian Cristina Pereira, Guilherme Ribeiro Romualdo

**Affiliations:** 1 Medical School, São Paulo State University (UNESP), Botucatu 18618-687, Brazil; taynara.vieira@unesp.br (T.A.V.); daniseloto@gmail.com (D.G.S.); joyce.rizzi@unesp.br (J.S.R.); paloma.peixoto@unesp.br (P.V.L.P.); betoni.correa@unesp.br (G.V.B.C.); juliana.siqueira@unesp.br (J.S.S.); nubia.grandini@unesp.br (N.A.G.); leticia.cardoso-valente@unesp.br (L.C.V.);; 2 Integrated Colleges of Bauru (FIB), Bauru 17056-100, Brazil; 3 Department of Pharmaceutical Sciences, University of Milan, 20133 Milan, Italy; giovanna.baron@unimi.it (G.B.); giancarlo.aldini@unimi.it (G.A.); 4 School of Agriculture Sciences, São Paulo State University (UNESP), Botucatu 18618-687, Brazil

**Keywords:** hepatic diseases, oxidative stress, antioxidants, flavonoids

## Abstract

Oxidative stress is involved in pathophysiological mechanisms associated with a myriad of liver diseases. Bergamot (*Citrus bergamia*) leaves yield a high level of antioxidant polyphenolic compounds that may hinder the development of liver diseases, while their potential is yet to be fully explored. Thus, the aim of the study was to test the effects of bergamot leaf extract (BLE) on hepatic and mitochondrial oxidative stress in different models. In vivo study: Wistar rats were distributed into two groups: control diet (C) and high-sugar–fat diet (HSF) for twenty weeks. Afterward, the animals were redivided to initiate a ten-week treatment with BLE: C, HSF, and HSF+BLE. In vitro study: Rat hepatic mitochondria were isolated by differential centrifugation and used to assess safety and efficacy of the BLE. Hepatocyte monolayer and spheroids were applied to evaluate the safety of physiologically plausible BLE concentrations and their effects on hydrogen peroxide-induced cytotoxicity. The results showed that BLE improved metabolic parameters, reduced hepatic triglyceride levels, malondialdehyde, and increased catalase activity in vivo. In vitro, BLE decreased lipid peroxidation and increased the ratio of reduced and oxidized glutathione in chemically challenged mitochondria. BLE did not exert cytotoxicity in the hepatocyte monolayer and spheroids, while attenuated oxidative stress-induced cytotoxicity. Data indicate that in vivo and in vitro hepatic oxidative stress is modulated by BLE, reinforcing that BLE may act as an agent against chronic liver diseases.

## 1. Introduction

Metabolic dysfunction-associated steatotic liver disease (MASLD) is a recently proposed concept to replace the term “non-alcoholic fatty liver disease (NAFLD)”. MASLD is a condition that comprehends the presence of hepatic steatosis and any of the following three metabolic conditions: overweight/obesity, diabetes mellitus, or evidence of metabolic dysregulation, even in lean individuals [1,2]. It is the most common liver disease, slow and progressive, which can progress to steatohepatitis, fibrosis, cirrhosis, and hepatocellular carcinoma [2].

The increased consumption of “westernized” foods, characterized by increased refined sugar and fat amounts, and sugar-sweetened beverages rich in fructose, has been contributing to MASLD development [2,3]. The associations between bad eating habits, genetics, and gut microbiota can result in an excess of free fatty acids and the accumulation of triglycerides in the hepatocytes, creating a lipotoxic environment that leads to negative hepatic outcomes, including inflammation, fibrosis, hepatocyte cell death, and oxidative stress [2].

Oxidative stress arises from an imbalance between the generation of reactive oxygen species (ROS) and antioxidant defenses. Mitochondria are the main sources of free radicals in cells, as they generate and sequester ROS, which can cause damage to many macromolecules, as protein, lipids, and nuclei acids [4]. The literature reports that oxidative stress is involved in processes associated with liver fibrosis. Furthermore, levels of antioxidant enzymes, such as glutathione (GSH) peroxidase, superoxide dismutase (SOD), and catalase (CAT), appear to be reduced in chronic liver disease, indicating an overall impairment of antioxidant capacity [5]. The generation of ROS may trigger lipid peroxidation, which is a chain reaction process where free radicals attack polyunsaturated fatty acids (PUFAs) in cell membranes, leading to the formation of lipid peroxides and secondary reactive aldehydes, as malondialdehyde (MDA), thereby sustaining and intensifying the oxidative insult in a self-perpetuating cycle [4,5]. Therefore, due to the significant involvement of oxidative stress in the pathophysiology of liver diseases, the application of antioxidant strategies has emerged as a potential tool to prevent and treat hepatic disorders [6,7].

Bergamot (*Citrus bergamia*) fruit has antioxidant and anti-inflammatory potential [8]. In addition, *C. bergamia* fruit demonstrated hypolipemic and hypoglycemic properties that have been shown to be effective in the treatment of metabolic syndrome [9,10,11]. These effects are mostly attributed to the polyphenolic content of this fruit, which is composed predominantly of flavonoids (e.g., naringin, neohesperidin, melitidin). Naringin is a flavanone glycoside composed of naringenin and the disaccharide neohesperidose. Neohesperidin is a flavanone glycoside structurally related to hesperidin, differing by the position of sugar attachment. Melitidin is a flavanone derivative with a statin-like moiety, featuring a 3-hydroxy-3-methylglutaryl (HMG) side chain [12,13,14]. However, recent investigations have shown, for certain types of plants, that leaves may yield higher concentrations of phenolic compounds compared to the fruit [14,15]. In the light of these findings, data from our group demonstrated that the leaves and fruits have a qualitative overlap of their polyphenol profiles, while the leaves may contain greater amounts of specific polyphenols. In further in vitro experiments, the leaves demonstrated higher antioxidant activity, while both components had similar anti-inflammatory effects [14].

Next, Siqueira et al. (2022) showed that this leaf extract was able to treat cardiorenal metabolic syndrome in obese rats [16]. Given these reports, studies using bergamot leaves to combat or prevent diseases due to oxidative stress are encouraged. Therefore, since there are no studies involving bergamot leaf extract (BLE) in in vivo and in vitro liver diseases, the aim of this study was to test the effects of BLE on hepatic and mitochondrial oxidative stress in different models.

## 2. Materials and Methods

### 2.1. Animal Ethics

All the procedures were approved by the local Animal Ethics Committee (1393/2021) and performed in accordance with the National Institute of Health’s Guide for the care and Use of Laboratory Animals [17].

### 2.2. BLE

The leaves were harvested on a farm in the Reggio Calabria region, Italy, and the dry extract was obtained at H&AD (Herbal & Antioxidant Derivatives S.r.l.) located in Località Chiusi, 89032 Bianco (RC), Italy. A study carried out by Baron et al. (2021), which characterized the bioactive compounds present in the extracts of bergamot leaves and fruit, showed that the leaf extract is particularly rich in polyphenols, such as flavones (luteolin, apigenin and chrysoeriol) and flavanones (neoeriocitrin, naringin, neohesperidin, melitidine, and brutieridine), and demonstrates superior antioxidant and anti-inflammatory activity compared to the fruit extract [14]. BLE was diluted in water or minimum essential medium (MEM) for in vivo or in vitro treatments.

### 2.3. In Vivo Study

In order to observe the effect of BLE on hepatic oxidative stress induced by diet male Wistar rats (n = 18, weighting around 180 g), 30 days old, were kept in an environmentally controlled room (22 ± 3 °C; 12 h light–dark cycle and relative humidity of 60 ± 5%) and randomly distributed into two groups to receive the control diet (C, n = 6 animals) or the high-sugar–fat diet plus 25% sucrose in water (HSF, n = 12 animals) for 20 weeks [18]. At the 20th week, after identifying significant differences in body weight (C: 464 ± 47 vs. HSF: 515 ± 59, *p* = 0.027) and plasma triglycerides levels (C: 84.5 ± 31.3 vs. HSF: 149.0 ± 24.1, *p* < 0.001) between the groups, animals were redistributed into 3 groups for BLE supplementation (50 mg/kg), namely control diet + placebo (C, n = 6 animals), high-sugar–fat diet + placebo (HSF, n = 6 animals), and high-sugar–fat diet + bergamot leaf extract (HSF+BLE, n = 6 animals) for 10 weeks. A daily dose of BLE (50 mg/kg body weight) was administered by gavage, and the placebo groups were given filtered water in the same manner. The diets and water were provided *ad libitum*. At the conclusion of the experimental period, animals were anesthetized with thiopental (120 mg/kg/IP) and euthanized by decapitation.

#### 2.3.1. Nutritional and Metabolic Parameters

Caloric intake, body weight and adiposity index were used to evaluate the nutritional profile. Calorie intake was calculated by multiplying the daily amount of food consumed (in grams) by the known caloric value of each macronutrient in the diet, based on its composition: 4 kcal/g for carbohydrates, 4 kcal/g for proteins, and 9 kcal/g for lipids. The total energy intake was thus determined from the sum of the caloric contributions of these macronutrients according to their respective proportions in the diet. For the HSF group, it also included calories from water (0.25 × 4 × mL consumed). The body weights were evaluated weekly. The adiposity index was used as an indicator of obesity through the formula described below using the fat deposits that were dissected after euthanasia. The sum of deposits were normalized by body weight [(epididymal + retroperitoneal + visceral)/body weight × 100] [16]. At the time of euthanasia, the liver was collected for oxidative stress analysis and intracellular triglyceride levels and blood was drawn to measure triacylglycerol levels by enzymatic colorimetric method using an automated device (Technicon, RA-XTTM System, Global Medical Instrumentation, Ramsey, MN, USA), using CELM^®^ kits, Barueri, São Paulo, Brazil, and a glucometer was used to measure glucose levels (Accu-Chek Performa; Roche Diagnostics, Indianapolis, IN, USA).

#### 2.3.2. Hepatic Triglycerides Levels

Liver tissue samples (approximately 100 mg) were homogenized (ULTRA-TURRAX^®^T25 basic IKA^®^ Werke, Staufen, Germany) with 1.0 mL of cold phosphate-buffered saline (PBS) at a pH of 7.4 and were centrifuged at 800× *g* at 4 °C for 10 min. The supernatant was used for the extraction of the lipid fraction in accordance with Cui et al. (2017) to determine intracellular hepatic triglyceride levels [19] using the colorimetric method (Triglyceride Colorimetric Assay Kit; Cayman Chemical, Ann Arbor, MI, EUA). The assay is based on the enzymatic hydrolysis of triglycerides to glycerol, followed by a series of reactions generating hydrogen peroxide, which reacts with a chromogen to produce a colorimetric signal proportional to triglyceride concentration. Absorbance was measured at 500 nm using a microplate reader. The results were adjusted based on tissue quantity.

#### 2.3.3. Oxidative Stress Markers and Antioxidant Enzymes Activity in Liver

MDA levels were used to estimate lipid peroxidation through reaction with thiobarbituric acid as previously described. They were expressed in nmol/g protein [20]. The activity of SOD was measured by spectrophotometry, based on the inhibition of the superoxide radical reaction with pyrogallol, and was expressed as U/mg protein [21]. CAT activity was evaluated by decreasing hydrogen peroxide (H_2_O_2_) levels. The breakdown of H_2_O_2_ in the reaction mixture was measured spectrophotometrically and expressed in pmol/mg protein [22].

### 2.4. In Vitro Study

#### 2.4.1. Mitochondrial Assays

##### Mitochondrial Isolation and Treatment

In order to monitor the protective capacity of the BLE on hepatic mitochondrial oxidative stress, male Wistar rats (n = 7), approximately 45 days old and weighing around 180 g, were acquired. The animals were kept in an environmentally controlled room (22 ± 3 °C; 12 h light–dark cycle and relative humidity of 60 ± 5%), four daily cycles of air exhaust, and a 12/12 h light/dark for around one week. Water and chow were *provided ad libitum*. After euthanization, hepatic mitochondria were isolated by differential centrifugation [23] as follows: the liver was rapidly removed, minced into approximately 50 mL of medium containing 250 mM of sucrose, 1 mM of ethylene glycol-bis (β-aminoethyl ether)-N,N,N′,N′-tetraacetic acid (EGTA), and 10 mM of HEPES potassium hydroxide buffer (HEPES-KOH), at a pH of 7.2 and at 4 °C, and was homogenized three times for 15 s at 1 min intervals in a Potter–Elvehjen homogenizer. The suspension was centrifuged at 770× *g* for 5 min and the resulting supernatant was centrifuged at 9800× *g* for 10 min. The pellet was resuspended with 10 mL of medium containing 250 mM of sucrose, 0.3 mM of EGTA, and 10 mM of HEPES-KOH, at a pH of 7.2, and was centrifuged at 4500× *g* for 15 min. The final mitochondrial pellet was suspended with 1 mL of medium containing 250 mM of sucrose and 10 mM of HEPES-KOH, at pH 7.2, and was utilized within 3 h.

Mitochondrial protein concentration was determined by the Biuret reaction. The absorbance was monitored in a spectrophotometer (Shimadzu CPS-240A, Shimadzu Corporation, Kyoto Japan) at λ 540 nm, using a calibration curve with bovine serum albumin as standard.

Extract aliquots diluted in water were prepared for in vitro evaluation in isolated liver mitochondria and exposed to BLE (10, 100, 250, and 500 µg/mL). The concentrations were established according to in vitro studies which utilized BLE [14,16]. Working solutions of bergamot leaf extract (BLE) were prepared by diluting stock aliquots in sterile distilled water to final concentrations of 10, 100, 250, and 500 µg/mL. Isolated liver mitochondria were then incubated with BLE at concentrations of 10, 100, 250, and 500 µg/mL in mitochondrial respiration buffer or an appropriate reaction medium for predetermined time periods, under gentle agitation and controlled temperature conditions. The exposure was carried out in microtubes or assay plates, ensuring a constant final volume and standardized mitochondrial protein concentration across all experimental groups. At the end of the incubation period, the mitochondrial preparations were subjected to specific analyses according to the previously defined functional and biochemical parameters.

##### Monitoring ROS Formation

The generation of ROS was observed spectrofluorimetrically by the Synergy HTX equipment (Biotek), with the use of the fluorescent probe cell-permeant 2′,7′-dichlorodihydrofluorescein diacetate (H_2_DCFDA) (Sigma, Kawasaki, Japan, Ref.: 4091-99-0) at wavelengths of 503 nm for excitation and 529 nm for emission. Mitochondria were incubated in standard reaction medium with 125 mM of sucrose, 65 mM of potassium chloride (KCl), and 10 mM of HEPES-KOH at pH 7.2, as well as 2.5 μM of rotenone and 2 μM of H_2_DCFDA, and were energized through the addition of 5 mM of potassium succinate. 2′-7′-dichlorodihydrofluorescein diacetate (DCFDA) is a molecule that, after crossing biological membranes, is hydrolyzed, releasing the non-fluorescent compound DCFH. However, in the presence of ROS, it is quickly oxidized and transformed into DCF, which presents a high quantum yield of fluorescence when excited at 503 nm. Thus, the increase in fluorescence is directly proportional to the production of RONS by mitochondria [24]. The tert-butyl hydroperoxide (t-BOOH) was used as a positive control due to its well-established ability to induce oxidative stress and cause cellular damage, particularly in mitochondria, through the generation of ROS.

##### Redox State of Pyridine Nucleotides (NAD(P)H) Evaluation

The redox state of NAD(P)H was monitored fluorometrically using the Synergy HTX equipment (Biotek) for 366 nm excitation and 450 nm for emission. Mitochondria were incubated in standard reaction medium containing 125 mM of sucrose, 65 mM of KCl, and 10 mM of HEPES-KOH, as well as 2.5 μM of rotenone [well-known inhibitor of NAD(P)H dehydrogenase (Complex I)], and were energized by the addition of 5 mM of potassium succinate. Reduced NAD(P)H are fluorescent, while oxidized NAD(P)^+^ are not fluorescent. Therefore, variations in fluorescence intensity indicate alterations in the redox state of NAD(P)H [25]. The t-BOOH was used as a positive control.

##### Mitochondrial Membrane Lipoperoxidation

Mitochondria were pre-incubated in 1 mL of medium containing 65 mM of KCl and 10 mM of HEPES-KOH at pH 7.2, as well as 2.5 μM of rotenone (Complex I inhibitor) and 5 mM of succinate (Complex II substrate). After 30 min, under stirring, 1 mL of thiobarbituric acid (TBA) (Millipore, Burlington, MA, USA, Ref.: 504-17-6) 1% (m/v) (prepared in sodium hydroxide (NaOH) 50 mM), 0.1 mL of NaOH 10 mM, and 0.5 mL of 20% phosphoric acid (m/v) were combined for 40 min at 94 °C. After cooling, the malondialdehyde-thiobarbituric acid complex (MDA-TBA) was extracted after the addition of 2 mL of N-butanol and after centrifugation at 2500× *g* for 2 min. The absorbance was measured at 535 nm and the MDA concentration, in nmoles/mg of protein, was calculated from ɛ = 1.56 × 10^5^ M^−1^cm^−1^ [26]. The ferro-citrate system (50 µM Fe^2^⁺ and 2 mM citrate) served as the positive control for membrane lipid peroxidation.

##### Reduced Glutathione (GSH) and Oxidized Glutathione (GSSG) Level Determination

The content of reduced glutathione in mitochondria was determined by fluorescence, as *o*-phthalaldehyde (OPT) (Sigma, Ref.: 643-79-8) reacts with GSH to form a highly fluorescent derivative. Fluorescence was measured at excitation and emission wavelengths of 350 nm and 420 nm, respectively [27]. Mitochondria were pre-incubated for 30 min at 30 °C with different concentrations of the test substance in a reaction medium containing 125 mM of sucrose, 25 mM of KCl, and 50 mM of HEPES-KOH at pH 7.2, in combination with with 5 mM of succinate, 2 μM of rotenone, and 10 μM of calcium chloride (CaCl_2_). After the incubation time, 0.5 mL of trichloroacetic acid (TCA) 13% (*w*/*v*) was added, and then the samples were centrifuged at 9000× *g* for 3 min, for protein precipitation. After centrifugation, aliquots of the supernatant were removed and added to a medium composed of 10 mM of sodium phosphate monobasic (NaH_2_PO_4_) and 5 mM of EGTA, at a pH of 8.0 and with 100 μL of OPT. Fluorescence was determined after 15 min on a Synergy HTX (Biotek, Charlotte, Vermont, USA), and GSSG levels were determined by adding 250 μL of N-ethyl-maleimide (NEM) 0.04 M to 250 μL aliquots of supernatant. After 20 min of incubation at room temperature, 500 μL of NaOH 10 M was added and 100 μL of this solution was added to 2 mL of NaOH 1 M and 100 μL of OPT. Fluorescence was determined after 15 min of incubation in the dark and the result determined by the GSH/GSSG ratio. The t-BOOH was used as a positive control.

### 2.5. Cell Culture Conditions, Treatments, and Viability

The human hepatoma cell line C3A/HepG2 (HB-8065, ATCC, Manassas, VA, USA) was cultured in MEM (Vitrocell, Campinas, Brazil) with low glucose, 1 mM of sodium pyruvate, 2 mM of L-glutamine, and 1% non-essential amino acids, supplemented with 10% fetal bovine serum (FBS) (Gibco, Waltham, MA, USA), 100 U/mL of penicillin, and 100 μg/mL of streptomycin (Gibco, USA) in conventional 96-well plates (5 × 10^3^ cells/mL, two-dimensional model, hepatocyte monolayer) or in ultra-low attachment u-shaped plates (174925, Thermo Fisher Scientific, Waltham, MA, USA) (5 × 10^4^ cells/mL, three-dimensional model, hepatocyte spheroids) [28]. Immediately (monolayer) or 48 h (spheroid formation time) after plate seeding, cells were treated with BLE in different physiologically plausible concentrations in the nanogram range (0, 0.01, 0.1, 1, 10, 100, 1000, 10,000, and 100,000 ng/mL) for 24 and 48 h to evaluate the safety of the extract in vitro, using a 3-(4,5-dimethylthiazol-2-yl)-2,5-diphenyltetrazolium bromide (MTT) viability assay. Next, using monolayers and spheroids, cells were challenged with 500 µM H_2_O_2_ [28] and/or treated with BLE (0, 0.1, 1, 10, and 100 ng/mL) for 24 and 48 h to evaluate BLE effects on oxidative stress-induced cytotoxicity, also using the MTT assay.

For all assays, the plates were incubated for 3 h with MTT solution (5 mg/mL) in a light-protected environment, the medium was completely removed from the wells, and the formazan crystals were dissolved by adding 100 μL of dimethyl sulfoxide (DMSO) per well. Absorbance was measured at 570 nm using an automated ELISA plate reader (Biotek 800T, Agilent, Santa Clara, CA, USA). All assays described included three biological replicates (subcultures) per treatment and three independent experiments.

### 2.6. Statistical Analysis

For in vivo study parameters, data were compared by one-way ANOVA followed by Tukey’s *post hoc* test for parametric data or by a Kruskal–Wallis test followed by Dunn’s *post hoc* test for non-parametric data. For the in vitro study, data were compared by one-way ANOVA followed by Dunn’s or Tukey’s test. The GraphPrism program (San Diego, CA, USA), version 5.0 for Windows, was used, and a significant difference was assumed considering *p* ≤ 0.05.

## 3. Results

### 3.1. In Vivo

#### 3.1.1. Nutritional and Metabolic Parameters: BLE Attenuated After 10 Weeks of Treatment the Metabolic Parameters

Table 1 shows the nutritional and metabolic parameters. The HSF group exhibited an increase in final body weight, caloric intake, adiposity index, plasma levels of triglycerides, and glucose, as opposed to the control group. On the other hand, the HSF group supplemented with BLE (HSF+BLE) showed an increase in caloric intake, adiposity index, glucose, and triglycerides compared to the control group, but a decrease in plasma levels of triglycerides and glucose compared to the HSF group.

#### 3.1.2. Hepatic Oxidative Stress Was Modulated by BLE

**Figure 1:**
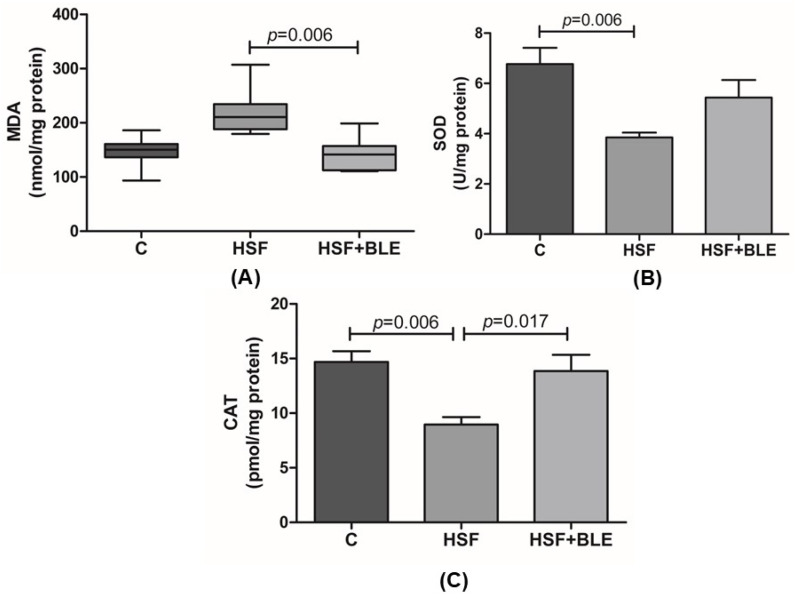
Antioxidant enzyme activities and lipid peroxidation in liver tissue after in vivo exposure. (**A**) Malondialdehyde (MDA); (**B**) superoxide dismutase (SOD); (**C**) catalase (CAT) activities. Control diet (C), high-sugar–fat diet (HSF) and high-sugar–fat diet supplemented with bergamot leaf extract (HSF+BLE) groups. Data are expressed as box plots or means + standard deviations, compared by one-way ANOVA followed by Tukey’s *post hoc* (CAT and SOD) or by the Kruskal–Wallis test followed by Dunn’s *post hoc* test (MDA). *p* ≤ 0.05 is significant.

Figure 1 illustrates that the HSF group exhibited a significant decrease in the activity of antioxidant enzymes SOD and CAT compared to the control group. However, BLE was able to reduce MDA levels and increase CAT activity in the HSF+BLE group compared to the HSF group.

#### 3.1.3. BLE-Modulated Hepatic Triglycerides Levels

**Figure 2:**
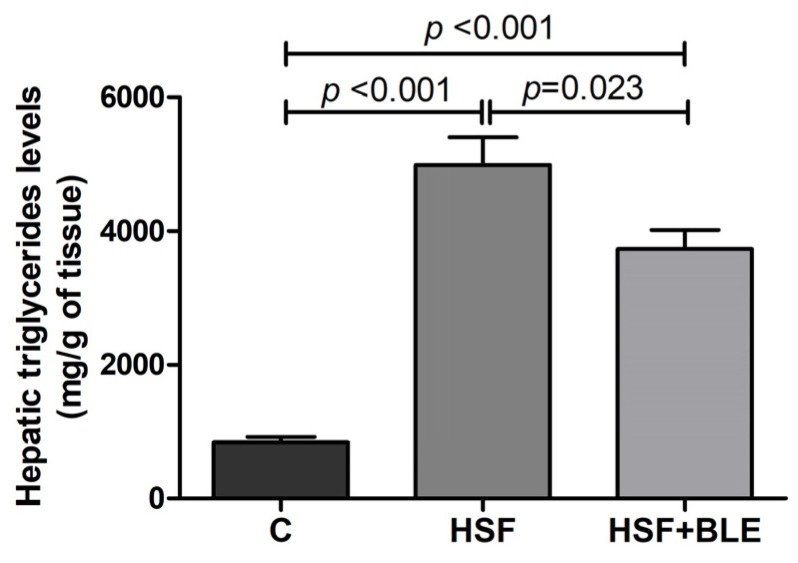
Hepatic triglyceride levels. Control diet (C), high-sugar–fat diet (HSF), and high-sugar–fat diet supplemented with bergamot leaf extract (HSF+BLE) groups. Data are expressed as means + standard deviations compared by one-way ANOVA followed by Tukey’s *post hoc* test. *p* ≤ 0.05 is significant.

Figure 2 shows hepatic triglyceride levels. The HSF group exhibited significantly higher levels than the control group, while the HSF+BLE group presented decreased levels in relation to the HSF group.

### 3.2. In Vitro

#### 3.2.1. BLE Has Antioxidant Capacity in Liver Mitochondria

**Figure 3:**
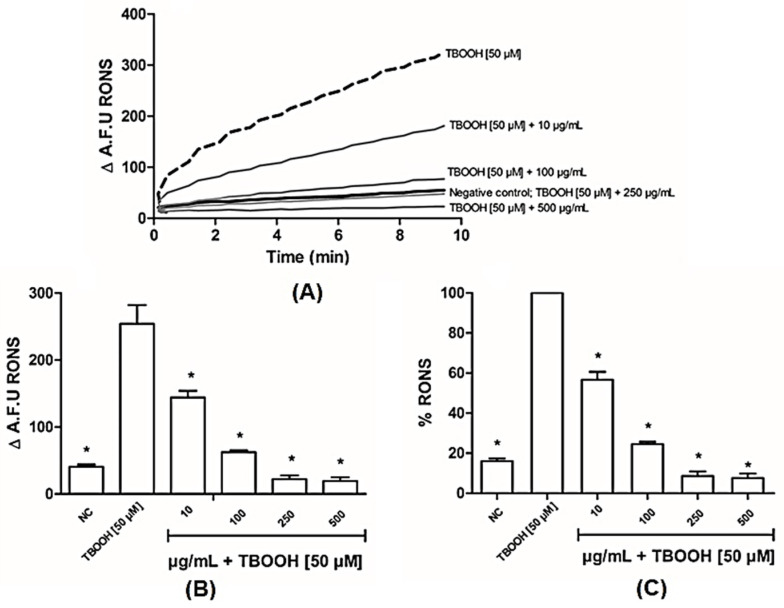
Effects of bergamot leaf extract (BLE) on reactive oxygen species (ROS) formation isolated mitochondria from young and healthy Wistar rats. (**A**) Tracing representatives of the formation and accumulation of ROS; (**B**) rate of ROS formation of the formation and accumulation of ROS; (**C**) percentage of accumulated ROS relative to total ROS formation. NC (negative control), A.F.U (arbitrary fluorescence unit). Data are expressed as means + standard deviations, with one-way ANOVA followed by Dunn’s post hoc test used to compare the concentrations and the positive control (TBOOH [50 µM]). (*) indicates significance at *p* ≤ 0.05, compared to the positive control.

The effect of induced oxidative damage can be seen in each graph with representative traces of tert-butyl hydroperoxide (TBOOH) exposure (Figure 3). Figure 3A–C shows the effects of BLE on protecting ROS formation, since all the tested concentrations of the extract were effective in reducing ROS formation induced by TBOOH.

**Figure 4:**
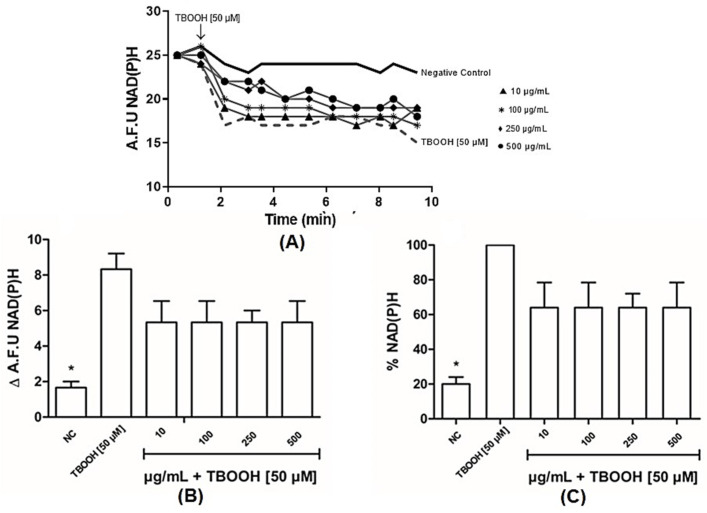
Effects of bergamot leaf extract (BLE) on NAD(P)H oxidation of mitochondria. (**A**) Representative trace of NAD(P)H oxidation; (**B**) NAD(P)H oxidation; (**C**) percentage of NAD(P)H oxidation. NC (negative control), A.F.U (arbitrary fluorescence unit). Data are expressed as means + standard deviations, with one-way ANOVA followed by Dunn’s post hoc test used to compare the concentrations and the positive control (TBOOH [50 µM]). (*) indicates significance at *p* ≤ 0.05, compared to the positive control.

Figure 4 shows NADPH oxidation increasing as well as a previous treatment of mitochondria with TBOOH. Our findings demonstrate that, even following exposure to varying concentrations of bergamot extract, the observed subtle protective effects were not significant. While NAD(P)H levels increased in mitochondria treated with TBOOH [50 µM], the administration of BLE, regardless of dosage, did not impact the oxidation of NAD(P)H in mitochondria.

**Figure 5:**
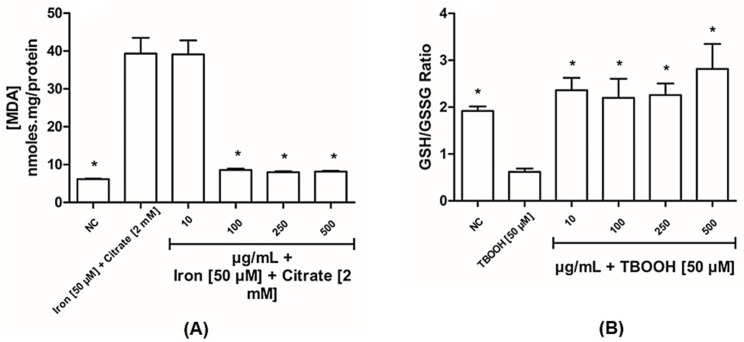
Effects of bergamot leaf extract (BLE) on the mitochondrial redox status. (**A**) Effects of bergamot leaf extract on the formation of thiobarbituric acid reactive species (TBARs); (**B**) effects of bergamot leaf extract on the GSH/GSSG antioxidant system. The positive control for the quantification of mitochondrial membrane lipoperoxidation was induced with 50 µM of iron and 2 mM of citrate, and the positive control for GSH/GSSG quantification was induced with TBOOH [50 µM]. NC (negative control). Data are expressed as means + standard deviations, with one-way ANOVA followed by Dunn’s *post hoc* test used to compare the concentrations and the positive control. (*) indicates significance at *p* ≤ 0.05, compared to the positive control.

To confirm the ability of BLE to protect liver mitochondria, lipid peroxidation by MDA-TBA complex and GSH/GSSG ratio were evaluated (Figure 5). The extract, in general, was able to protect the mitochondria. However, the lowest concentration (10 µg/mL) of the extract was not able to prevent membrane lipid peroxidation by the ferro-citrate system.

#### 3.2.2. BLE Does Not Reduce Cell Viability and Attenuates Oxidative Stress-Induced Cytotoxicity

**Figure 6:**
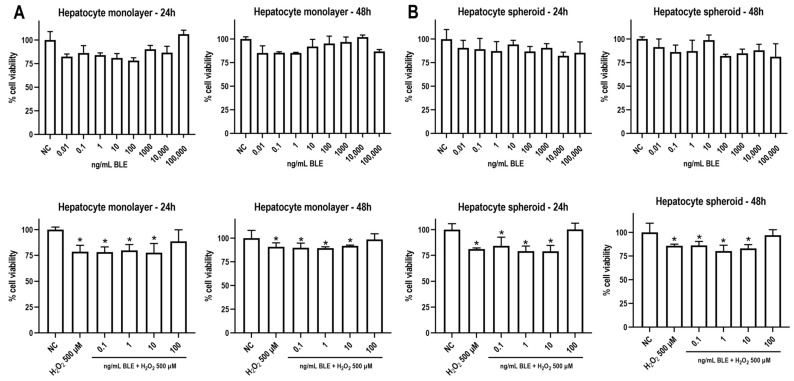
Effects of bergamot leaf extract (BLE) on hydrogen peroxide (H_2_O_2_)-induced cytotoxicity in (**A**) 2D and (**B**) 3D hepatocyte models. Monolayers were exposed to H_2_O_2_ (500 µm) to induce cytotoxicity and were simultaneously treated with BLE at 0.1, 1, 10, and 100 ng/mL for 24 or 48 h. NC (negative control). Data are expressed as means + standard deviations, compared using one-way ANOVA followed by Tukey’s *post hoc* test. (*) indicates significance compared to NC and/or 100 ng/mL at *p* ≤ 0.05.

As BLE increased the antioxidant capacity in the liver mitochondria at the microgram (µg) level, we further investigated whether even lower concentrations in nanograms (ng)—closer to a physiologically plausible exposure—would attenuate H_2_O_2_-induced cytotoxicity in 2D and 3D hepatocyte models. As shown in Figure 6, a wide range of BLE concentrations (0.01–100,000 ng/mL) did not exert significant cytotoxicity in both human hepatocyte monolayer and spheroids, regardless of the timepoint, indicating the safety of the extract in terms of cell viability. Some of these concentrations (0.1, 1, 10, and 100 ng/mL) were chosen for the next experiments. It is of note that 100 ng/mL of BLE was able to prevent H_2_O_2_-induced cytotoxicity in both models at both timepoints. In keeping with these data, at higher concentrations—in the µg range—BLE did not impair cell viability or proliferation and reduced T-BOOH-induced oxidative stress in hepatocytes (Supplementary Material, Figure S1).

## 4. Discussion

Given the scarcity of studies on bergamot leaves in hepatic models, both in vivo and in vitro, the aim of this study was to test the effects of bergamot leaf extract on hepatic and mitochondrial oxidative stress in different models. It is well established in the literature that the consumption of high-sugar–fat diets can not only trigger obesity and related disorders, such as hypertriglyceridemia but can also affect and compromise the function of other organs, such as the liver [29,30,31].

Our in vivo results corroborate the existing literature, as the HSF developed by our research group could lead to obesity, hypertriglyceridemia, increased hepatic triglycerides, and the decreased activity of antioxidant enzymes in liver tissue compared to the control group. In contrast, supplementation with bergamot leaf extract improved glucose levels, dyslipidemia, lipid peroxidation, and hepatic triglyceride levels and also increased the activity of the antioxidant enzyme catalase in the liver.

Numerous studies have emphasized a strong association between flavonoid-rich diets and improvements in metabolic diseases [32,33,34]. In particular, studies with *Citrus bergamia*, also known as bergamot, have shown significant anti-hyperglycemic and hypocholesterolemic effects, as well as antioxidant/free radical scavenging activities. Moreover, this fruit has garnered significant interest because of its flavonoid profile, since it contains flavanones that can act as natural statins [9,12]. Another study carried out with bergamot juice observed an attenuation of dyslipidemia and hepatic oxidative stress in rats fed with a high-fat diet [35].

It has been reported that naringin specifically, a flavonoid component of BLE, is known to exert a protective action on oxidative damage in tert-butyl hydroperoxide-induced HepG2 injury [36]; in addition, it has demonstrated anti-inflammatory, antioxidant, and anti-apoptotic activities on H_9_C_2_ cells and macrophages [37,38,39]. Furthermore, other studies have demonstrated that the flavonoids found in bergamot have a positive impact on inflammation, decreasing the expression of pro-inflammatory cytokines in the liver and potentially increasing the gene expression of anti-inflammatory markers [40]. And the results obtained in this study are in agreement with those found in the fruit; therefore, we can infer that both the fruit and leaves of *Citrus bergamia* have an impact on metabolic parameters and hepatoprotective effects. Within this context, it can be said that the leaves of this plant can also be used in the treatment of diseases with the advantage of being a sustainable product regardless of harvest.

Regarding the effects of bergamot on mitochondrial redox state parameters, we found a few studies that evaluated these parameters with *Citrus bergamia* fruit and none with leaf extract. Algieri et al. (2022) [41] evaluated the effects of bergamot polyphenolic fraction (BPF) on the mitochondrial bioenergetics of porcine aortic endothelial cells (pAECs) exposed to doxorubicin (DOX). Results showed the inhibition of mitochondrial activity with 10 µM of DOX, which persisted without improvement with 200 µg/mL of BPF. On the other hand, the decrease in basal respiration and adenosine triphosphate (ATP) production caused by 0.5 or 1.0 µM of DOX was improved in 100 or 200 µg/mL of BPF; these parameters were not evaluated in this study. Other studies demonstrated that bergamot fruit can prevent intracellular RONS accumulation and the decreased cell death of human neuroblastoma (SH-SY5Y) induced by N-Methyl-D-aspartic acid (NMDA) [42]. In line with these findings, this study illustrates that BLE acts as an antioxidant, since it has shown protective effects on in vitro oxidative stress assays using hepatic mitochondria isolated from rats. These data are promising, as protection against reactive species found in hepatic mitochondria may contribute to our understanding of the results obtained by Musolino et al. (2020) [10]. Parafati et al. (2015) [43] found that bergamot fruit had beneficial effects on MASLD, as reactive species are part of the pathophysiology of this disease and a protective effect on mitochondria may have contributed to improving the pathophysiological factors of MASLD. 

It is well established that oxidative stress can be caused by excessive ROS, such as peroxides and superoxide. Excessive ROS can stimulate the oxidative stress state in cells, which can lead to the oxidative damage of cell macromolecules, such as DNA, proteins, and lipids [44]. In the experiment carried out to observe the effects of BLE on NAD(P)H oxidation in mitochondria, Figure 4A shows a significant drop in NAD(P)H when mitochondria were treated with TBOOH [50 µM], which is well explained by the activation of the glutathione peroxidase/reductase system, which is able to metabolize the spiked hydroperoxides. In particular, glutathione peroxidase reduces hydroperoxides to the corresponding alcohols, a redox reaction based on GSH as a substrate that is oxidized to GSSG, which in turn is reduced by glutathione reductase, a reaction that consumes NADPH [45]. Bergamot did not significantly change at any tested dose the NAD(P)H content, suggesting that it does not affect the enzymatic detoxification of the hydroperoxides.

When DCFH_2_, an oxidizable substrate, was spiked to mitochondria in the presence of TBOOH, a time-dependent oxidative conversion to the corresponding fluorescent product was observed, as shown in Figure 3A. The rate of DCFH_2_ oxidation is reported in Figure 3 and this oxidative process is explained by taking into consideration that a fraction of TBOOH escapes from the enzymatic reducing defense and decomposes to form radical species which induce a time-dependent cascade of oxidative reactions. Bergamot was found to dose-dependently reduce the oxidation rate, thus indicating a direct radical scavenging towards the radical species generated by the decomposition of the hydroperoxide. As expected, TBOOH spiking also induced a significant drop in GSH and a corresponding increase in the oxidized form, GSSG, leading to a significant reduction in the GSH/GSSG ratio. Bergamot was found to significantly inhibit GSH oxidation and the GSH/GSSG ratio was found to be even higher than in control samples. The results can be explained by taking into consideration that GSH is heavily oxidized in the oxidizing milieu induced by TBOOH and that bergamot is effective in preventing GSH consumption by reducing the radical-mediated oxidation process. Complementing these data, a reduction in the lipid peroxidation of the mitochondrial membranes was observed from the dose of 100 µg/mL, reinforcing the antioxidant potential of bergamot leaves. Baron and colleagues [14] showed that the leaves have a greater antioxidant capacity compared to the fruit, confirming our results. A potential mechanism could be the stimulation of the nuclear factor erythroid 2-related factor 2 (NRF-2), which was identified by Siqueira and colleagues [16] *in vitro*. Furthermore, it is already known that extracts from various types of leaves have antioxidant capacities and can be used as therapeutic adjuvants in various diseases.

Enhanced oxidative stress can also culminate in hepatocyte death, which is closely involved in the progression of many liver diseases, including MASLD. During MASLD pathogenesis, lipotoxicity and oxidative stress contribute to organelle impairment and inflammatory processes, which in turn initiate a cascade of cell death in the liver [46]. A growing body of evidence indicates that different hepatocyte death mechanisms (apoptosis, necroptosis, pyroptosis, and ferroptosis) have the potential to be effective targets in preventing and treating MASLD [46]. Our in vitro findings in hepatocyte monolayers and spheroids—well-accepted preclinical and translational models [47]—demonstrated that BLE may protect against oxidative stress-induced cytotoxicity, which a promising target for MASLD emergence.

## 5. Conclusions

These data allow us to conclude that in vivo and in vitro hepatic oxidative stress are modulated by bergamot leaf extract. Therefore, BLE can be considered a novel herbal medicine that acts against the repercussions of oxidative stress on liver tissue.

## Figures and Tables

**Table 1:** Nutritional and metabolic parameters.

	Groups				Effect	
	C	HSF	HSF+BLE	C	C	HSF
				*vs*	*vs*	*vs*
				HSF	HSF+BLE	HSF+BLE
Final body weight (g)	489 ± 57	610 ± 74 *	562 ± 66	0.017	0.170	0.444
Caloric intake (kcal/day)	90.0 ± 6.0	104.9 ± 8.7 *	101.7 ± 8.3 *	0.013	0.049	0.766
Adiposity index (%)	4.18 ± 1.15	9.13 ± 1.38 *	7.65 ± 1.94 *	<0.001	0.004	0.248
Triglycerides (mg/dL)	26.2 ± 4.7	108.4 ± 9.9 *	60.5 ± 11.9 *^,#^	<0.001	<0.001	<0.001
Glucose (mg/dL)	81.0 ± 4.5	94.5 ± 3.0 *	86.5 ± 5.0 *^,#^	<0.001	0.014	0.097

Control diet (C), high-sugar–fat diet (HSF), and high-sugar–fat diet supplemented with bergamot leaf extract (HSF+BLE) groups. Data are expressed as means ± standard deviations, compared by one-way ANOVA followed by Tukey’s post hoc test. *p* ≤ 0.05 is significant. * vs. C; ^#^ *vs* HSF.

## Data Availability

The data that support the findings of this study are available from the corresponding author upon reasonable request.

## Supplementary Materials

The following supporting information can be downloaded at: https://www.mdpi.com/article/10.3390/antiox14050543/s1. References [48,49,50,51] are cited in the Supplementary Materials.

## Abbreviations

BLE	Bergamot leaf extract
C	Control diet
HSF	High-sugar–fat diet
HSF+BLE	High-sugar–fat diet + bergamot leaf extract
MASLD	Metabolic dysfunction-associated steatotic liver disease
NAFLD	Non-alcoholic fatty liver disease
ROS	Reactive oxygen species
DNA	Deoxyribonucleic acid
GSH	Reduced glutathione
SOD	Superoxide dismutase
CAT	Catalase
MDA	Malondialdehyde
PUFAs	Polyunsaturated fatty acids PUFAs
RNS	Reactive nitrogen species
H&AD	Herbal & Antioxidant Derivatives S.r.l.
RC	Province of Reggio Calabria
MEM	Minimum essential medium
USA	United States of America
PBS	Phosphate-buffered saline
H_2_O_2_	Hydrogen peroxide
EGTA	Ethylene glycol-bis (β-aminoethyl ether)-N,N,N′,N′-tetraacetic acid
HEPES-KOH	HEPES–potassium hydroxide buffer
RONS	Reactive oxygen/nitrogen species
H_2_DCFDA	2′,7′-dichlorodihydrofluorescein diacetate
KCl	Potassium chloride
DCFDA	2′-7′-dichlorodihydrofluorescein diacetate
NAD(P)H	Nicotinamide adenine dinucleotide phosphate hydrogen
TBA	Thiobarbituric acid
NaOH	Sodium hydroxide
GSSG	Oxidized glutathione
OPT	*o*-phthalaldehyde
CaCl_2_	Calcium chloride
TCA	Trichloroacetic acid
NaH_2_PO_4_	Sodium phosphate monobasic
NEM	N-ethyl-maleimide
FBS	Fetal bovine serum
MTT	3-(4,5-dimethylthiazol-2-yl)-2,5-diphenyltetrazolium bromide
DMSO	Dimethyl sulfoxide
TBOOH	Tert-butyl hydroperoxide
NC	Negative control
A.F.U	Arbitrary fluorescence unit
BPF	Bergamot polyphenolic fraction
pAECs	Porcine aortic endothelial cells
DOX	Doxorubicin
ATP	Adenosine triphosphate
NMDA	N-Methyl-D-aspartic acid
NRF-2	Nuclear factor erythroid 2-related factor 2
