# Supplementary material for: Bergamot Leaf Extract as an Agent Against Chronic Liver Diseases? In Vitro and In Vivo Findings on Oxidative Stress Modulation
Source: Antioxidants (Basel). 2025 Apr 30;14(5):543. doi: 10.3390/antiox14050543 (PMC12108294; doi:10.3390/antiox14050543)
Supplement: Supplementary file 1 [file antioxidants-14-00543-s001.zip › antioxidants-3597812-supplementary.docx]

**SUPPLEMENTARY MATERIAL**

1. **Methods**
   1. *HepG2 cell culture exposed to BLE*

Human hepatocellular carcinoma cell line (HepG2) was obtained from the Cell Bank of Rio de Janeiro (BCRJ, Rio de Janeiro, code 0103, lot: 001025) and was cultured in low-glucose DMEM (Dulbecco’s Modified Eagle Medium, Sigma-Aldrich, USA) buffered at pH 7.2 with 1.5 g/L sodium bicarbonate, 10.000 U/mL penicillin-streptomycin and 10% fetal bovine serum (FBS) (Gibco, USA). Cells were maintained at 37 °C in a 5% CO_2_ atmosphere and assays were performed in culture flasks at 80% confluence with a limit of 15 passages after thawing. BLE was dissolved in Milli-Q water at 10, 100, 250 and 500 µg/mL, concentrations corresponding to those evaluated in isolated mitochondria. Assays were evaluated after 24 and 48 hours of exposure to BLE. All tests included three biological (subculture) and four technical replicates.

*1.2 MTT cell viability assay*

The MTT assay assesses cell viability by converting yellow tetrazolium salt (3-(4,5- Dimethylthiazol-2-yl)-2,5-diphenyl tetrazolium bromide (MTT), Sigma Aldrich, USA) into purple formazan crystals via mitochondrial and cytoplasmic enzymes [1]. HepG2 cells were plated in 96-well plates at a density of 2 x 10^4^ cells. After adhesion, they were treated with BLE concentrations and the positive control CCCP (10 µM, carbonyl cyanide m- chlorophenylhydrazone, CAS 555-60-2, Sigma Aldrich, USA) and incubated for 24 and 48 hours. Post-incubation, the culture medium was removed, and phenol-free DMEM with 10% MTT (5 mg/mL in PBS) was added. After 3 hours at 37 °C, the MTT solution was removed, and formazan crystals were dissolved in DMSO and 10% glycine buffer (0.2 M, pH 10.2). Absorbance was measured at 570 nm using a Synergy HTX spectrofluorometer (Biotek), with results expressed as a percentage relative to the negative control.

*1.3 Sulforadomine B assay cell proliferation*

The SRB assay measures cell proliferation by quantifying protein content using sulforhodamine B dye [2]. HepG2 cells were plated at a density of 10^5^ cells and treated after adhesion. After the incubation period, the cells were washed and fixed with a methanol/acetic acid solution. After this process, the cells were stained with sulforhodamine B. Finally, the cells were washed with a 1% acetic acid solution to remove excess dye. The dye bound to the cells was then solubilized with a Tris, and the absorbance was measured at 540 nm using a Synergy HTX spectrofluorometer (Biotek). The results were expressed as a percentage relative to the negative control (culture medium only).

*1.4 RONS production in HepG2 Cells*

The production of RONS in cells was analyzed using the CM-H_2_DCFDA (5,6- chloromethyl-2’,7’-dichlorodihydrofluorescein diacetate, acetyl ester) probe, which becomes fluorescent DCF when oxidized [3]. HepG2 cells were plated (10^5^ cells per well), treated, with the PC and concentrations (except the negative control) exposed to T-BOOH (200 µM, CAS 75-91-2, 458139, Sigma-Aldrich,USA) for 45 minutes to induce ROS production. After the incubation periods, the cells underwent trypsinization and centrifugation, after which the pellet was resuspended in phenol-free DMEM with the CM-H_2_DCFDA probe (2 mM in DMSO). The incubation was followed by reading in Synergy HTX spectrofluorometer (Biotek) with an excitation wavelength of 503 nm and an emission wavelength of 528 nm. RONS production was normalized to protein mass quantified by the Bradford method [4]. To facilitate comparison of the protective effects and in consideration of the induction of oxidative stress in the PC and BLE concentrations, the data have been expressed as a percentage in relation to the PC.

1. **Results**

*BLE does not affect cell viability, induced cell proliferation and was able to protect cells when oxidative stress was induced*

BLE does not affect negatively cell viability and proliferation in preliminary assays and reduces the state of oxidative stress in HepG2 cell culture. Supplementary Figure 1A shows that exposure to BLE did not affect cell viability. Both at 24 and 48 hours, the exposure of cells to different concentrations of BLE does not impair the metabolic capacity of the cells. In the same line, Supplementary Figure 1B shows that the exposure of BLE does not impair proliferation of HepG2 cells. Supplementary Figure 1C reports RONS protection when oxidative stress was induced by T-BOOH 100 µM. All studied concentrations were effective in protecting and preventing oxidative damage. The data reflect that BLE was effective in protection against RONS production, also, that RONS production at 48 hours was lower compared to 24 hours. Additionally, the higher the concentration, the greater the protection.


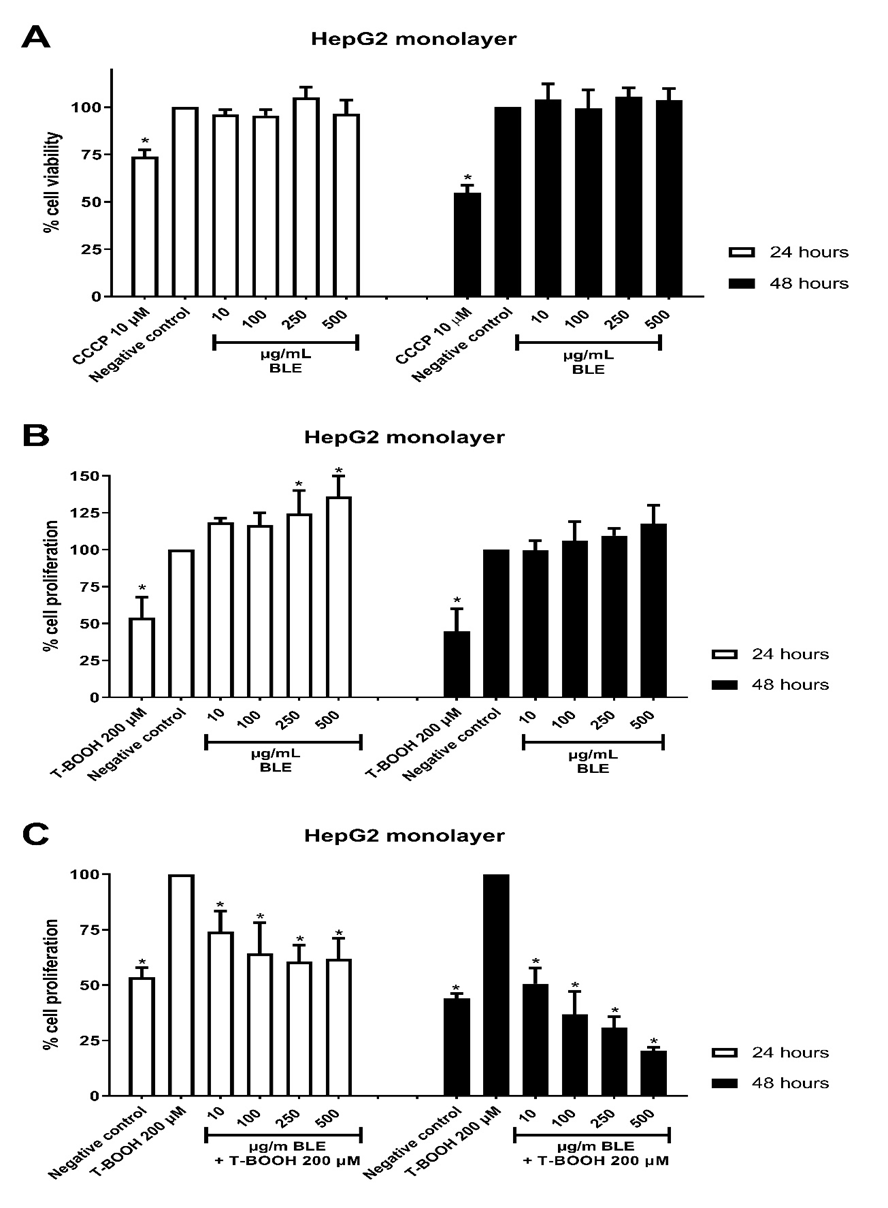


**Supplementary Figure 1.** Effects of bergamot leaves extract (BLE) on cell viability in a hepatocyte model. HepG2 cells were treated with concentrations of 10, 100, 250, and 500 µg/mL for 24 and 48 hours. The negative control (NC) was culture medium only, and the positive control (PC) was CCCP 10 µM (**A**). Effects of BLE on cell proliferation in a hepatocyte model. PC was T-BOOH 200 µM (exposure for 2 hours) (**B**). Data are expressed as a percentage relative to NC (100%) as mean ± standard deviation, compared by ANOVA followed by Dunnett’s post hoc test, (*) significant compared to NC for p ≤ 0.05. (**C**) Effects of BLE on protection against oxidative stress induced by exposure to T-BOOH 100 µM (exposure for 45 minutes). HepG2 cells were treated with concentrations of 10, 100, 250 and 500 µg/mL for 24 and 48 hours and subsequently exposed to T-BOOH 100 µM (exposure for 45 minutes), along with the PC. Data are expressed as a percentage relative to PC (100%) as mean ± standard deviation, compared by ANOVA followed by Dunnett’s *post hoc* test, (*) significant compared to PC for p ≤ 0.05.

1. **References**

1. Kumar, P.; Nagarajan, A.; Uchil, P.D. Analysis of Cell Viability by the MTT Assay. *Cold Spring Harb. Protoc.* **2018**, *2018*, pdb.prot095505, doi:10.1101/pdb.prot095505.

2. Vichai, V.; Kirtikara, K. Sulforhodamine B Colorimetric Assay for Cytotoxicity Screening. *Nat. Protoc.* **2006**, *1*, 1112–1116, doi:10.1038/nprot.2006.179.

3. Chernyak, B. V.; Izyumov, D.S.; Lyamzaev, K.G.; Pashkovskaya, A.A.; Pletjushkina, O.Y.; Antonenko, Y.N.; Sakharov, D. V.; Wirtz, K.W.A.; Skulachev, V.P. Production of Reactive Oxygen Species in Mitochondria of HeLa Cells under Oxidative Stress. *Biochim. Biophys. Acta - Bioenerg.* **2006**, *1757*, 525–534, doi:10.1016/j.bbabio.2006.02.019.

4. Bradford, M. A Rapid and Sensitive Method for the Quantitation of Microgram Quantities of Protein Utilizing the Principle of Protein-Dye Binding. *Anal. Biochem.* **1976**, *72*, 248–254, doi:10.1006/abio.1976.9999.
